# Comparison of TachoSil and TachoComb in patients undergoing liver resection—a randomized, double-blind, non-inferiority trial

**DOI:** 10.1007/s00423-017-1571-z

**Published:** 2017-04-01

**Authors:** S. Kawasaki, H. Origasa, V. Tetens, M. Kobayashi

**Affiliations:** 1 Department of Hepatobiliary Pancreatic Surgery, Juntendo University Graduate School of Medicine, Tokyo, Japan; 2 Division of Biostatistics and Clinical Epidemiology, University of Toyama Graduate School of Medicine and Pharmaceutical Sciences, Toyama City, Japan; 3 Clinical Science, Takeda Development Centre Europe Ltd, London, UK; 4 R&D Japan, CSL Behring K.K, 1-7-12 Shinonome, Koto-ku, Tokyo, 135-0062 Japan

**Keywords:** Hemostasis, Japan, Liver, Safety, TachoComb, TachoSil

## Abstract

**Background:**

This study aimed to demonstrate the noninferior efficacy of TachoSil vs. TachoComb in Japanese patients undergoing liver resection and to assess the safety of TachoSil vs. TachoComb in these patients.

**Methods:**

This randomized, double-blind, noninferiority study (JapicCTI-090684) involved participants scheduled for liver resection/living donors (age ≥ 20 years). TachoSil or TachoComb (1:1 allocation ratio) was applied to control persistent exudative bleeding after primary hemostasis during liver resection/removal for donation. The primary outcome was hemostasis 5 min after study treatment application. The 95% confidence interval (CI) for the difference in the proportion of participants with hemostasis 5 min after application of TachoSil/TachoComb was determined; noninferiority of TachoSil was indicated if the lower limit of the CI was ≥−14%. Adverse events (AEs) were recorded.

**Results:**

All participants in the efficacy analysis (TachoSil: 54/54, 100%; TachoComb: 54/54, 100%) achieved hemostasis 5 min after study treatment application. Therefore, TachoSil was noninferior to TachoComb. All participants experienced ≥1 AE; however, none discontinued because of an AE. Most (≥97.8%) AEs were mild or moderate in severity.

**Conclusions:**

These findings confirm the safety profile and noninferior hemostatic efficacy of TachoSil compared with TachoComb.

## Introduction

Hemostasis during liver resection is a critical determinant of surgical success. Indeed, the volume of blood loss during liver surgery is an established predictor of morbidity and mortality [1–3]. Of note, the management of hemorrhage during liver resection can be particularly challenging compared with other types of surgery because the hepatosinusoidal structure lacks smooth muscle and thus the capacity to vasoconstrict [4]. As a consequence, a variety of different approaches may be used to manage bleeding during liver resection. Major bleeding (strong and/or pulsating) from identifiable vessels is initially managed by primary surgical methods (i.e., sutures, stapling ligatures, argon beam coagulation, and electrocautery) and then secondary hemostatic agents as necessary [5, 6]. Diffuse bleeding may be managed by argon beam coagulation, electrocautery, and/or hemostatic agents, including collagen-based sealants, synthetic glues, and fibrin sealants [5–8]. Of these hemostatic agents, fibrin sealants have been increasingly used for hemostasis during liver resection [9], in which they may be used to control persistent bleeding (after primary surgical management) and/or diffuse bleeding.

TachoComb® and TachoSil® are widely used fibrin sealants for tissue adhesion/closure during different types of surgery, including liver, lung, cardiovascular, gynecological, and urological. TachoComb comprises a collagen patch coated with human fibrinogen and bovine thrombin and aprotinin. This product version has been marketed and widely used in Japan since 1999. The newer product version, TachoSil, is currently marketed in more than 50 countries worldwide, and it comprises a collagen patch coated with human fibrinogen and human thrombin. TachoSil was developed after TachoComb to avoid potential immunogenic effects of bovine thrombin [10] and anaphylaxis caused by bovine aprotinin with repeated use [11, 12], and to negate the theoretical risk of horizontal disease transfer (bovine to human), e.g., by prions causing variant Creutzfeldt–Jakob disease. Preclinical testing in pigs demonstrated that there was no difference between TachoSil and TachoComb in primary hemostasis and that aprotinin was not required for effective hemostasis [13]. Subsequent clinical studies [14–16] carried out in Europe demonstrated that TachoSil provides effective hemostasis during liver resection and has an acceptable safety and tolerability profile. To date, however, no comparison of the efficacy and safety of the two product versions has been published to demonstrate if the exclusion of aprotinin has an impact on clinical outcomes. Likewise, the hemostatic efficacy of TachoSil has not previously been investigated in Asians (including Japanese) with their different coagulation profile compared with Caucasians [17].

Accordingly, the aims of this study were to demonstrate noninferiority in efficacy of TachoSil compared with TachoComb in Japanese patients undergoing liver resection and to assess and compare the safety of the two products in these patients.

## Methods

### Study design

This was a multicenter, randomized, double-blind, noninferiority (TachoSil vs. TachoComb) study carried out at 11 sites in Japan from 18th April 2008 to 19th August 2009. The trial was registered in the Japan Pharmaceutical Information Center Clinical Trial Information database (JapicCTI-090684).

The study protocol was reviewed and approved by the Institutional Review Board at each study site. The study was carried out in accordance with Good Clinical Practice (GCP), based on the ethical principles outlined in the Declaration of Helsinki and the International Conference on Harmonization-GCP Guideline, and all applicable local Japanese laws and regulations. All participants provided written informed consent.

### Study population

Patients scheduled for elective liver resection (at least segmental, anatomical, or non-anatomical) and living donors were considered for inclusion in the study if they were aged ≥20 years at the time of informed consent.

Patients and living donors were excluded from the study if they had a Child–Pugh Classification of C; had a history of hypersensitivity to any of the ingredients of the investigational products, or to bovine blood-derived, bovine lung-derived, or equine blood-derived preparations; had a history of TachoComb use; were females who were pregnant, lactating, suspected of being pregnant, or who planned to become pregnant during the study period; had participated in any other clinical study within 180 days before providing informed consent; or were judged by the investigator as not suitable to participate in the study.

### Randomization and treatment protocol

Prior to surgery, participants were randomly allocated (1:1 ratio) to TachoSil or TachoComb using a central allocation system. Randomization was stratified by study site, Child–Pugh classification (A or B), and platelet count (<100,000 or ≥100,000/μL). Intra-operatively, patients with severe surgical complications and patients with persistent bleeding after completion of the primary hemostatic procedures were excluded from the study.

TachoSil and TachoComb were provided (Nycomed, Zurich, Switzerland [now part of Takeda Pharmaceutical Company Ltd]) in the form of identical 9.5 × 4.8 cm equine collagen patches of a 0.5 cm thick spongy material with a dry yellow coating of active ingredients on one side. Active ingredients in the TachoSil patches were human fibrinogen (5.5 mg/cm^2^) and human thrombin (1.38 IU/cm^2^). Active ingredients in the TachoComb patches were human fibrinogen (5.5 mg/cm^2^), bovine thrombin (1.38 IU/cm^2^), and bovine aprotinin (128 IU/cm^2^). The two study treatments were indistinguishable in their appearance and physical characteristics.

Study treatment was applied at the discretion of the investigator/subinvestigator to control persistent bleeding after primary hemostasis during liver resection or liver removal for donation. Before application, the wound site was cleansed of blood and body fluid. The patch was cut to the required dimensions and applied in dry form, or moistened with physiological saline just before application. The active side was applied to the bleeding wound site and held against the site with gentle pressure for 5 min.

To prevent potential confounding of the efficacy and safety evaluations, participants were prohibited from taking the following concomitant medications from 2 days before study treatment until day 28 after study treatment: tissue sealants containing fibrinogen, collagen preparations, oxidized cellulose preparations, local hemostatic agents, whole blood and platelet preparations, fresh frozen plasma, procoagulants, antifibrinolytic agents, aprotinin preparations, and other investigational drugs or medical devices.

### Outcome measures

The primary efficacy outcome was hemostasis 5 min after the application of study treatment, which was assessed at the site where study treatment was first applied. Blood flow occlusion was temporarily released, if clamping was used, and the site was visually inspected by the investigator/subinvestigator. Hemostasis was defined as being achieved when other hemostatic treatment was not required. If hemostasis was not obtained 5 min after application, other (nonstudy) treatments were used to control bleeding from the wound.

Adverse events (AEs) were recorded from the time of informed consent until day 28 after treatment and were defined as any unfavorable medical occurrence or worsening of subjective symptoms/objective findings, worsening of underlying diseases and complications, or clinically significant abnormal laboratory values. Adverse events were coded according to MedDRA, version 11.0.

Serological tests for hepatitis B and C virus (HBV and HCV) and human immunodeficiency virus (HIV) were performed the day before study treatment and at day 28.

### Statistical analysis

Based on the results from European clinical studies of patients undergoing liver resection, 95% of Japanese participants were expected to achieve hemostasis at 5 min. A sample size of 100 participants (50 per study group) was determined to provide 90% power at a noninferiority margin of 0.14.

The efficacy analysis population for demonstrating non-inferiority was the per protocol set and included all enrolled participants who received the allocated study treatment and from whom any data was collected (full analysis set), in accordance with the intention-to-treat principle [18]. The safety analysis population included all enrolled participants who received the allocated study treatment and provided any data.

The primary efficacy analysis, designed to demonstrate noninferiority of TachoSil compared with TachoComb, was the 95% confidence interval (CI) for the difference between TachoSil and TachoComb in the proportion of participants with hemostasis 5 min after application of TachoSil or TachoComb. Noninferiority of TachoSil was considered to be demonstrated if the lower limit of the 95% CI was greater than −14%. The 95% CI for the proportion of participants with hemostasis at 5 min in each group was also calculated using the score method by Wilson [19]. The difference in the proportion of participants with hemostasis at 5 min between the TachoSil and TachoComb groups was compared using Fisher’s exact test.

Safety analysis comprised the number of AEs, the number of participants who experienced AEs, and the number of participants who were HBV, HCV, and HIV positive at day 28.

## Results

### Disposition of participants

**Fig. 1:**
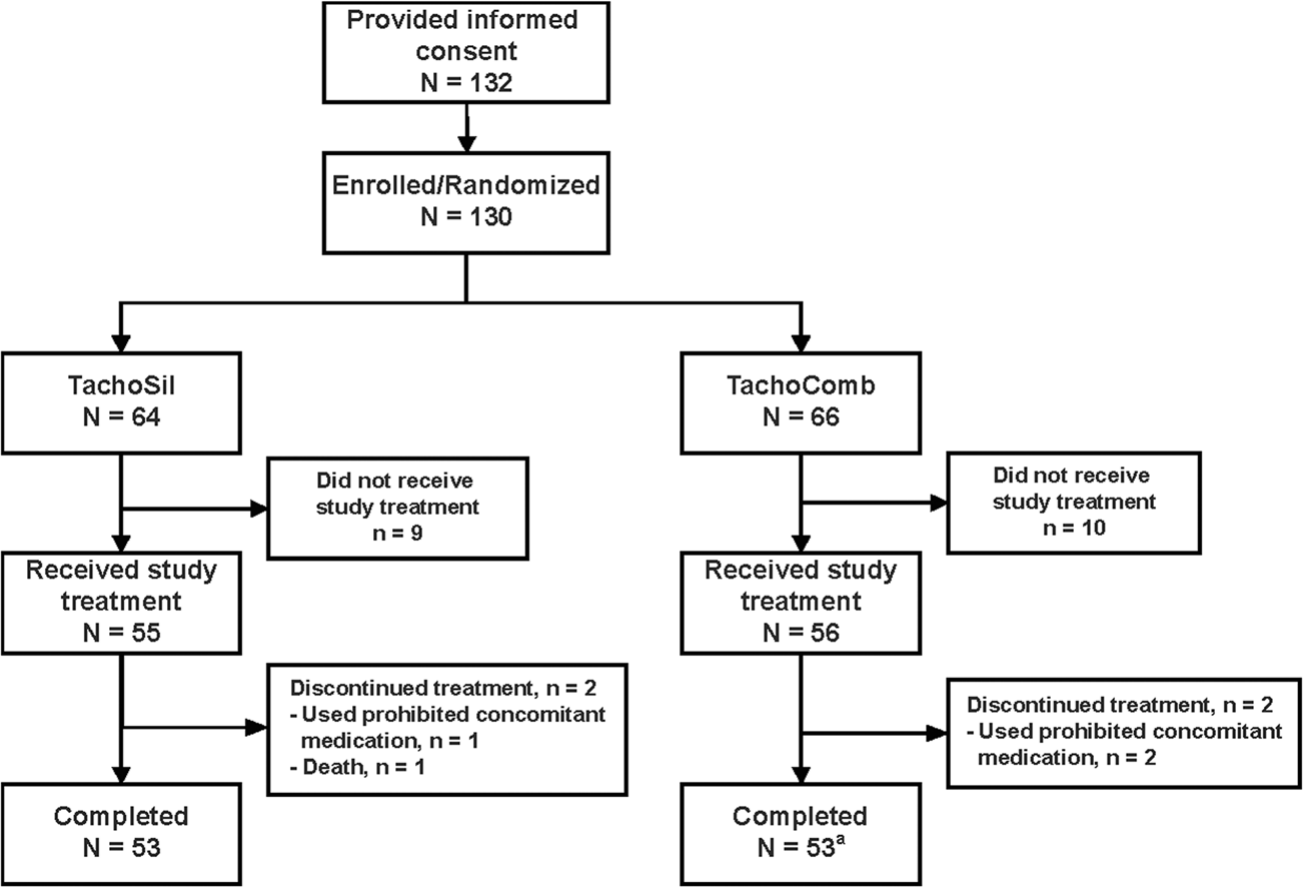
Disposition of participants. One participant did not discontinue treatment, but did not complete the day 28 examination (*a*)

A total of 130 participants were randomized to treatment, 64 to TachoSil and 66 to TachoComb (Fig. 1). Of the 64 participants randomized to TachoSil, 55 (85.9%) received study treatment, two (3.1%) discontinued from study participation, and 53 (82.8%) completed the study. Of the 66 participants randomized to TachoComb, 56 (84.8%) received study treatment, two (3.0%) discontinued from study participation, and 53 (80.3%) completed the study (note: one participant in this group completed the study, but did not complete the day 28 examination). Except for one participant in the TachoSil group who died due to intra-abdominal hemorrhage, all other participants who were discontinued had prohibited concomitant medications.

The efficacy analysis population included 54 participants in both study groups. The safety analysis population included 55 participants in the TachoSil group and 56 participants in the TachoComb group.

### Demographic, baseline, and surgical characteristics

Demographic and baseline characteristics were generally similar between study groups (Table 1), although there were more alcohol users (37.0 vs. 14.8%) and fewer patients undergoing surgery due to liver donation (22.1 vs. 3.7%) in the TachoSil group compared to the TachoComb treatment group. Overall, surgical characteristics were balanced between TachoSil and TachoComb treatment groups, respectively, with small differences in the use of drip bipolar (14.8 vs. 20.8%) and clamp crushing technique (13.0 vs. 7.4%) for hepatic resection (Table 2). Likewise, there were differences in the proportion of patients receiving intraoperative blood transfusion. More participants had a history of chemotherapy for hepatic disease in the TachoSil group compared with the TachoComb group.

**Table 1:** Participants’ demographic and baseline characteristics

Characteristic	TachoSil *N* = 54	TachoComb *N* = 54
Males, *n* (%)	37 (68.5)	35 (64.8)
Age (years), mean ± SD	59.9 ± 12.2	65.5 ± 11.3
BMI (kg/m^2^), mean ± SD	23.7 ± 3.2	22.6 ± 2.8
Smokers, *n* (%)	20 (37.0)	8 (14.8)
Alcohol use, *n* (%)	23 (42.6)	20 (37.0)
Reason for surgery, *n* (%)		
Hepatic disease	48 (88.9)	52 (96.3)
Liver donor	6 (11.1)	2 (3.7)
Child–Pugh classification, *n* (%)		
A	53 (98.1)	52 (96.3)
B	1 (1.9)	2 (3.7)
Platelets (×10^4^/μL), mean ± SD	20.7 ± 9.1	19.0 ± 6.7
Previous chemotherapy for hepatic disease, *n* (%)	19 (35.2)	8 (14.8)

*BMI* body mass index, *SD* standard deviation

**Table 2:** Participants’ surgical characteristics

Characteristic	TachoSil *N* = 54	TachoComb *N* = 54
Method of liver resection, *n* (%)		
CUSA	44 (81.5)	42 (77.8)
Drip bipolar	8 (14.8)	11 (20.4)
Harmonic scalpel	9 (16.7)	9 (16.7)
Clamp crush	7 (13.0)	4 (7.4)
Bipolar scissors	4 (7.4)	5 (9.3)
Other	20 (37.0)	15 (27.8)
Number of resection sites, mean ± SD	2.6 ± 1.40	3.1 ± 1.5
Weight of liver resected (g), mean ± SD	315.3 ± 274.2	338.4 ± 302.1
Intraoperative blood transfusion, *n* (%)		
Before efficacy evaluation	0 (0)	3 (5.6)
After efficacy evaluation	12 (22.2)	8 (14.8)
Blood flow occlusion, *n* (%)	47 (87.0)	49 (90.7)
Method of primary hemostasis, *n* (%)		
Suture	18 (33.3)	18 (33.3)
Ligation	13 (24.1)	17 (31.5)
Electrocoagulation	43 (79.6)	45 (83.3)
Compression	23 (42.6)	19 (35.2)
Volume of bleeding (mL), mean ± SD	649.5 ± 932.2	666.3 ± 499.0
Size of patch used, *n* (%)		
9.5 × 4.8 cm	9 (16.7)	11 (20.4)
4.8 × 4.8 cm	5 (9.3)	1 (1.9)
2.4 × 4.8 cm	6 (11.1)	4 (7.4)
2.4 × 2.4 cm	21 (38.9)	27 (50.0)
Other	13 (24.1)	11 (20.4)

*CUSA* Cavitron ultrasonic surgical aspirator, *SD* standard deviation

### Primary outcome measure

All participants in the efficacy analysis population in both study groups (TachoSil: 54/54, 100%; TachoComb: 54/54, 100%) achieved hemostasis 5 min after the application of study treatment. There was no difference in the response rate between study groups (*p* = 1.0). The 95% CI for the proportion of participants who achieved hemostasis at 5 min was 93.4 to 100% for both groups. The 95% CI for the difference in the proportion of participants who achieved hemostasis at 5 min between study groups was −4.9 to 4.9%, indicating that TachoSil was noninferior to TachoComb.

### Safety

The proportion of participants who experienced AEs was generally similar between study groups (Table 3). All participants in both study groups experienced at least one AE; however, no participants discontinued because of an AE. Most (≥97.8%) AEs in both groups were classified as mild or moderate in severity. The incidence of possibly treatment-related AEs was greater in the TachoSil group than in the TachoComb group.

**Table 3:** Summary of adverse events

Adverse event	TachoSil *N* = 55	TachoComb *N* = 56
One or more AE, *n* (%)	55 (100.0)	56 (100.0)
One or more possibly treatment-related AE, *n* (%)	10 (18.2)	1 (1.8)
Discontinuation because of an AE, *n* (%)	0 (0)	0 (0)
One or more SAE, *n* (%)	7 (12.7)	9 (16.1)
Total AEs, *n*	329	303
Mild, *n* (%)	263 (79.9)	232 (76.6)
Moderate, *n* (%)	59 (17.9)	65 (21.5)
Severe, *n* (%)	7 (2.1)	6 (2.0)

*AE* adverse event, *SAE* serious adverse event

A small number of participants experienced at least one serious AE (SAE), most of which were not considered to be related to study treatment. In the TachoSil group, seven participants experienced a total of eight SAEs, including ventricular flutter, peritonitis, intra-abdominal hemorrhage, pyrexia, post-procedural bile leak, atelectasis, aneurysm ruptured, and vascular pseudoaneurysm (all *n* = 1). The SAEs of post-procedural bile leak and peritonitis were considered to be possibly treatment related. The participant who experienced intra-abdominal hemorrhage died; this SAE was not considered to be related to study treatment because the event was a rupture caused by hepatic artery aneurysm resulting from the surgery. In the TachoComb group, nine participants experienced a total of 10 SAEs, including post-procedural bile leak (*n* = 4), and ventricular fibrillation, gastritis, abdominal abscess, upper limb fracture, chylothorax, and arterial hemorrhage (all *n* = 1). The SAE of ventricular fibrillation was considered to be possibly treatment related. One participant in the TachoComb group died after the study period due to worsening of underlying disease; this death was not considered to be related to study treatment.

The incidence of AEs (by preferred term) was generally similar between study groups (Table 4), with wound complication (defined as pruritus of the wound and periwound skin, wound pain, wound site pain, or wound pain in the buttocks) and pyrexia being the most commonly reported AEs, experienced by ≥73.2% of participants in both study groups.

**Table 4:** Adverse events occurring at a rate of >5%

Adverse event (preferred term), *n* (%)	TachoSil *N* = 55	TachoComb *N* = 56
Wound complication^a^	51 (92.7)	47 (83.9)
Pyrexia	44 (80.0)	41 (73.2)
Insomnia	17 (30.9)	12 (21.4)
Vomiting	11 (20.0)	8 (14.3)
Nausea	10 (18.2)	10 (17.9)
Abdominal distension	9 (16.4)	6 (10.7)
Musculoskeletal pain	9 (16.4)	5 (8.9)
Pleural effusion	8 (14.5)	14 (25.0)
Constipation	8 (14.5)	9 (16.1)
Ascites	6 (10.9)	5 (8.9)
Diarrhea	5 (9.1)	8 (14.3)
Hyperglycemia	5 (9.1)	5 (8.9)
Atelectasis	5 (9.1)	5 (8.9)
Pain	4 (7.3)	2 (3.6)
Blood pressure increased	4 (7.3)	1 (1.8)
Post-procedural bile leak	3 (5.5)	8 (14.3)
Back pain	3 (5.5)	4 (7.1)
Pruritus	3 (5.5)	3 (5.4)
Urine output decreased	3 (5.5)	2 (3.6)
Erythema	3 (5.5)	2 (3.6)
Rash	3 (5.5)	2 (3.6)
Cholangitis	2 (3.6)	5 (8.9)
Delirium	1 (1.8)	7 (12.5)
Abdominal abscess	0 (0)	3 (5.4)

^a^Pruritus of the wound and periwound skin, wound pain, wound site pain, or wound pain in the buttocks

Changes in laboratory values were similar between groups (data not shown). One participant in the TachoSil group experienced a clinically significant, but nonserious, abnormal laboratory value of C-reactive protein increase that was considered related to study treatment. There were no new cases of HBV, HBC, or HIV infection, nor were there any positive conversions for participants who had positive virus markers at the start of the study.

## Discussion

This is the first randomized trial to compare the efficacy and safety of TachoSil and TachoComb in Japanese patients undergoing liver resection or living donors. We found that TachoSil has noninferior hemostatic efficacy compared with TachoComb and appears to have a similar safety profile. Our findings therefore suggest that the beneficial effects of TachoSil are similar to those of TachoComb and that TachoSil is an effective means of providing hemostasis in Japanese patients during surgery.

The primary finding of our study was that TachoSil and TachoComb provided similarly effective hemostasis within 5 min of application in all participants. The assessment of hemostasis was judged by the surgeon; however, the binary nature of the endpoint avoids any grading and thus renders some objectivity. This endpoint reflects the immediate pharmacodynamic effect of the products, and although of minor clinical relevance, this kind of efficacy endpoint is widely used and acknowledged as basis for the assessment of hemostatic effect of treatments in surgery [4, 15, 20–22]. To our knowledge, no other published clinical study has directly compared the hemostatic efficacy of TachoSil and TachoComb. However, our findings are supported by those from nonclinical studies. [23] Notably, Agger et al. [13] found that TachoSil and TachoComb had similar hemostatic efficacy in a pig model of challenging aortic arterial bleeding. The findings from the current study support other published data showing that TachoSil is an effective means of providing hemostasis during liver resection. Specifically, two European, multicenter, randomized, controlled trials, involving 121 [16] and 119 [14] adults, respectively, demonstrated that TachoSil provided superior or significantly faster hemostasis compared with argon beamer during liver resection. Likewise, a US, multicenter, randomized, controlled trial, involving 224 adults, demonstrated that TachoSil provided superior hemostasis to Surgicel® during liver resection [20]. A small prospective study [15] involving 16 children undergoing liver resection also demonstrated that 81.3% of participants had effective hemostasis within 3 min of application of TachoSil. Further, TachoSil has also been reported to provide effective hemostasis during various other types of surgery, including lung surgery [24, 25], kidney tumor resection [22], prostate surgery [26], gynecological surgery [27–29], and cardiovascular surgery in patients requiring cardiopulmonary bypass [21]. The current published literature on use of TachoSil in Japan is restricted to a recently published retrospective study [30], a case report [31], and a case series [32]. In the retrospective study, which involved 75 patients, the application of TachoSil or TachoComb to the staple line of the pancreas after distal pancreatectomy was associated with a low rate of fistula formation [30]. The case report [31] and case series [32] describe successful pulmonary and renal application, respectively.

TachoSil was developed to reduce the potential for immunogenic effects in response to bovine thrombin and bovine aprotinin with repeated use, and to remove the potential for horizontal disease transfer (bovine to human). While no specific antibody testing was undertaken in the current analyses, a recent study reported that among 97 patients using TachoSil for the secondary treatment of local bleeding after hepatic resection, immunogenicity findings did not appear to be clinically significant [20]. No treatment emergent AEs related to immune response were reported in the initial 6-month follow-up period, and no medical conditions potentially related to antibody development were observed in an extended follow-up of up to 2 years.

We did not aim to assess the long-term safety of TachoSil in this study; however, the safety profile of TachoSil in clinical practice has been confirmed in postmarketing settings [33], through an estimated 5.2 million patient exposures worldwide (data on file). The findings from randomized controlled trials of TachoSil carried out in Europe, involving participants undergoing liver resection [14, 16] and other types of surgery [21, 22, 24], have also confirmed the favorable safety profile of TachoSil. Our study findings indicate that the safety profiles of TachoSil and TachoComb are similar for up to 28 days following use for hemostasis in Japanese patients undergoing liver resection or living donors. Treatment-related AEs were more common in the TachoSil group than in the TachoComb group. This difference likely relates to a random imbalance in treatment assignment to two investigators, both of whom practiced standards for the evaluation of the definition of “causal relationship” differently from the other investigators. All other aspects of safety including total AEs and patients with one or more SAEs were similar in the two treatment groups. Most AEs were of mild or moderate severity and few SAEs were reported. The incidence of AEs in our study is also higher than the incidence of AEs in the European studies of patients undergoing liver resection (100% of participants vs. 42 to 44% of participants [14, 16]). We suggest that this disparity likely reflects methodological differences in the prespecified criteria for recording or not recording specific AEs or the cautious practice of Japanese investigators. In particular, AEs that are common after liver resection, such as wound complication, nausea, vomiting, and pain, were recorded in our study, but not in the European studies.

A key strength of this study is the prospective, randomized, double-blind, multicenter design with predefined statistical analyses based on the intention-to-treat paradigm. Limitations include the length of follow-up, which may have been insufficient to detect longer term postoperative morbidities, and the moderate number of participants. Additionally, one of the authors (M. Kobayashi) was sponsor-affiliated, which incurs a risk of bias [34]. However, the primary contributors to the study design and the scientific interpretation of the data were the authors affiliated with academic research institutions.

## Conclusion

This study demonstrated that TachoSil has noninferior hemostatic efficacy compared with TachoComb, and a similar safety profile, in patients undergoing liver resection.
